# Outcome and prognostic factors in cervical cancer patients treated with surgery and concurrent chemoradiotherapy: a retrospective study

**DOI:** 10.1186/s12957-017-1307-0

**Published:** 2018-01-29

**Authors:** Yan-Mei Liu, Ling-Qin Ni, Sai-Sai Wang, Qian-Ling Lv, Wei-Jun Chen, Shen-Peng Ying

**Affiliations:** 1 Department of Radiation Oncology, Tai Zhou Central Hospital, Tai Zhou, Zhe Jiang 318000 China; 2 Department of Obstetrics and Gynecology, Tai Zhou Central Hospital, Tai Zhou, Zhe Jiang 318000 China

**Keywords:** Cervical carcinoma, Postoperative radiotherapy, Chemoradiotherapy, Prognosis

## Abstract

**Background:**

The objective of this study is to analyze the treatment outcome and secondary reactions in 98 patients with stage I–III cervical carcinoma who underwent postoperative radiotherapy.

**Methods:**

From 2006 to 2014, 98 patients with stage I–III cervical carcinoma were treated with postoperative radiotherapy. The major histological type, found in 92.86% of the patients (91 cases), was squamous cell carcinoma. Patients were staged according to the 2002 TNM guidelines. The postoperative radiotherapy methods included two-field irradiation (16 patients, 16.32%), four-field box irradiation (16 patients, 16.32%), and intensity-modulated radiotherapy (IMRT; 66 patients, 67.36%). The survival rates were represented using Kaplan-Meier curves, and prognosis analyses were performed using Cox multivariate analyses.

**Results:**

The 5-year overall survival and progression-free survival rates were 82.0 and 76.0%, respectively. Only one patient (1.02%) developed a grade 3 acute radiation enteritis, while grade 3 and 4 myelosuppression was noted in 17 patients (17.35%) and one patient (1.02%), respectively. Multivariate analyses showed that anemia before radiotherapy and tumor size were predictors of the OS (*P* = 0.008, *P* = 0.045) rates.

**Conclusions:**

Postoperative radiotherapy for patients with risk factors of cervical cancer procured good efficacy levels with mild side effects. Anemia and tumor size were important OS predictors.

## Background

As the third most frequent cancer among women worldwide, cervical cancer is the fourth leading cause of cancer deaths in women [1]. According to the global cancer statistics of 2012, 528,000 new cases of cervical cancer were diagnosed each year and 266,000 patients died just in 2012 [2]. According to previous reports, most of the cervical cancer patients in the early stage (IB to IIA) who have accepted proper treatment experienced positive outcome, with only 10 to 15% of the patients with risk factors experiencing recurrence [3]. Therefore, it is very important for cervical cancer patients to receive timely, accurate diagnosis and appropriate treatment to reduce the chances of recurrence.

The mechanism of action of cisplatin involves the formation of platinum-DNA adducts. The mechanisms underlying the interaction between chemotherapy and radiotherapy may include inhibition of the tumor’s impaired damage repair systems and an increase in the radiosensitivity of hypoxic cells. Compliance with treatment is very important for local control and overall survival (OS) in patients with cervical carcinoma.

Adjuvant chemoradiotherapy has been shown to improve the progression-free and overall survival outcome for patients with positive surgical margins, positive lymph nodes, or involvement of the parametrium [4]. Radiotherapy (RT), when delivered in a postoperative setting, has been associated with an increased risk of treatment-related toxicity, including gastrointestinal (GI), genitourinary, and lymphatic system toxicities compared with patients undergoing RT alone [5]. This study was conducted to evaluate the outcome and the prognostic factors of postoperative cervical cancer after concurrent chemoradiotherapy (CCRT).

## Methods

The present study retrospectively analyzes previous clinical trial results involving patients with cervical cancer who had undergone postoperative radiotherapy in the Radiation Oncology Department of the Tai Zhou Central Hospital. All patients had signed informed consent prior to entering the trials.

### Patient population

All the materials were obtained with agreement of patients and signed informed consent. The usage of human materials for analysis was approved by the ethical committee of Tai Zhou Central Hospital.

A total of 98 patients who underwent surgical resection for cervical cancer with at least one high-risk factor, such as primary tumor diameter ≥ 40 mm, deep invasion, lymphovascular space invasion (LVSI), and so on, were enrolled in this study between 2006 and 2014. The age of enrolled patients ranged from 29 to 76 years old, the mean age being 52. Postoperative pathological staging was performed according to the 2002 guidelines of the Federation International of Gynecology and Obstetrics (FIGO).

### Evaluation

Postoperative radiotherapy was performed approximately 4 weeks after radical surgery. Prior to the treatment protocol, each patient underwent physical, laboratory, and radiological examinations. Laboratory examinations included a complete blood cell count, measurement of liver and renal functions, and electrocardiography. All of the abovementioned indexes met the radiotherapy standard before the start of the treatment.

### Treatments

#### Radiotherapy

External whole pelvis irradiation using a 6- or 15-MV photon beam was performed with a dose of 1.8 Gy per fraction five times per week for a total dose of 45 Gy. Of the 98 patients, 16 were irradiated using the anteroposterior parallel opposing technique, 16 with four-field box irradiation, and 66 with intensity-modulated radiotherapy (IMRT).

#### Chemotherapy

Of the 98 patients, 71 underwent CCRT, which was given according to one of the three following cisplatin-based regimens: (i) weekly cisplatin (40 mg/m^2^) in 66 patients, (ii) weekly nedaplatin (35 mg/m^2^) in two patients, and (iii) carboplatin (AUC = 2) plus paclitaxel (135 mg/m^2^) every 3 weeks in three patients.

#### Toxicity

Clinical data regarding treatment-related complications were also collected. Complications occurring within 90 days from starting the primary treatment were considered as acute, and those occurring later than 90 days were considered as late complications. The severity of acute complications was classified according to the NCI Common Terminology Criteria for Adverse Events, Version 2.0. Late complications were graded according to the Radiation Therapy Oncology Group (RTOG) Late Radiation Morbidity Scoring Scheme [6].

### Follow-up evaluations

After treatment completion, the patient’s follow-ups were scheduled at 1 month, then every 3 months for the first 2 years, and every 6 months thereafter. Follow-ups included a physical examination, tumor marker detection, and pelvic imaging. Central or parametrial pelvic failure was defined as disease persisting or recurring in the pelvis. Distant failure was defined as a disease occurring outside of the pelvis, including the paraaortic lymph nodes.

### Statistical analysis

OS and progression-free survival (PFS) analyses were performed using the Kaplan–Meier method. Differences in survival were compared using the log-rank statistical test. Survival prognostic factor analyses were performed using the Cox regression method. Hazard ratios were given with 95% confidence intervals (95% CI). SPSS software 22.0 was used for statistical analyses. A *P* value < 0.05 was considered statistically significant.

## Results

### Patient characteristics

The patients and tumor-associated characteristics were summarized in Table 1. Seven patients (7.14%) were older than 70 years old. The major histological subtype of the tumors was squamous cell carcinoma (92.86%). The distribution of TNM stage among the patients was as follows: stage I, 32.65%; stage II, 29.59%; and stage III, 37.76%. In addition, 43.88% of the patients presented bulky tumors (larger or equal to 4 cm), 37.76% of the patients were lymph-node-positive, and 27.55% were anemic before the radiotherapy.

**Table 1:** Patients’ characteristics (*n* = 98)

Characteristic	*n* (%)
Age (years)	
< 40	8(8.16)
40–50	32(32.65)
51–60	34(34.69)
61–70	17(17.35)
> 70岁	7 (7.14)
Histology	
Squamous	91(92.86)
Adenosquamous	4(4.08)
Other type	3(3.06)
TNM stage (2002)	
I	32(32.65)
II	29(29.59)
III	37(37.76)
Pathological risk factors	
LVSI	37(42.85)
Tumor diameter ≥ 4 cm	43(43.88)
Deep stromal invasion	43(43.88)
LNM	37(37.76)
Parametrial involvement	4(4.08)
Anemia before radiotherapy	27(27.55)
Perineural invasion	24(24.49)

岁: Proportion of cervical cancer patients in all age groups by the stratification of each age

### Survival

All patients completed the radiotherapy treatment as planned. More than three cycles of weekly cisplatin were administered to 62 patients, and nedaplatin was administered to two patients. Two cycles of tri-weekly chemotherapeutic agents in combination were administered to two patients. In the presence of grade 3 myelosuppression, concurrent chemotherapy was suspended and the whole treatment was delayed while maintaining supportive care. After resolution, the chemotherapeutic agent dosage was reduced by 20% in the next cycle. In case of hepatic or renal toxicity, chemotherapy was suspended.

**Fig. 1:**
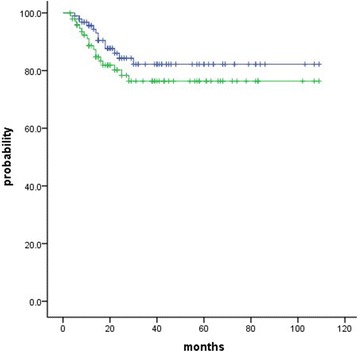
Survival curves of overall and progression-free survival

The median follow-up time was 37 months (range 6–118 months). At the time of analysis, 80 patients were alive without evidence of disease and five patients were alive with disease and 12 patients died of cervical cancer and one patient died of another cause. The 5-year OS and PFS rates were 82.0 and 76.0%, respectively (Fig. 1).

**Fig. 2:**
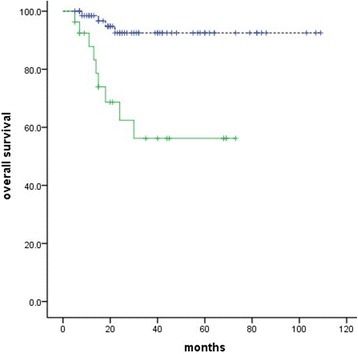
Effect of anemia on overall survival (*P* = 0.00)

**Fig. 3:**
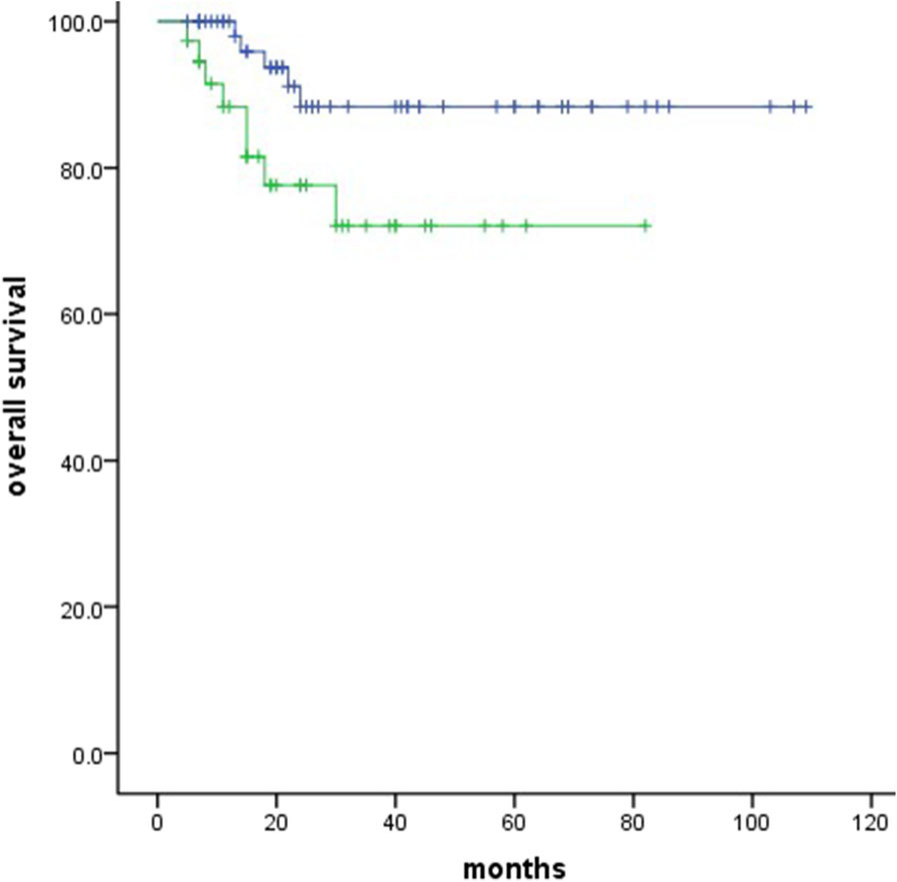
Effect of lymph node status on overall survival (*P* = 0.04)

**Fig. 4:**
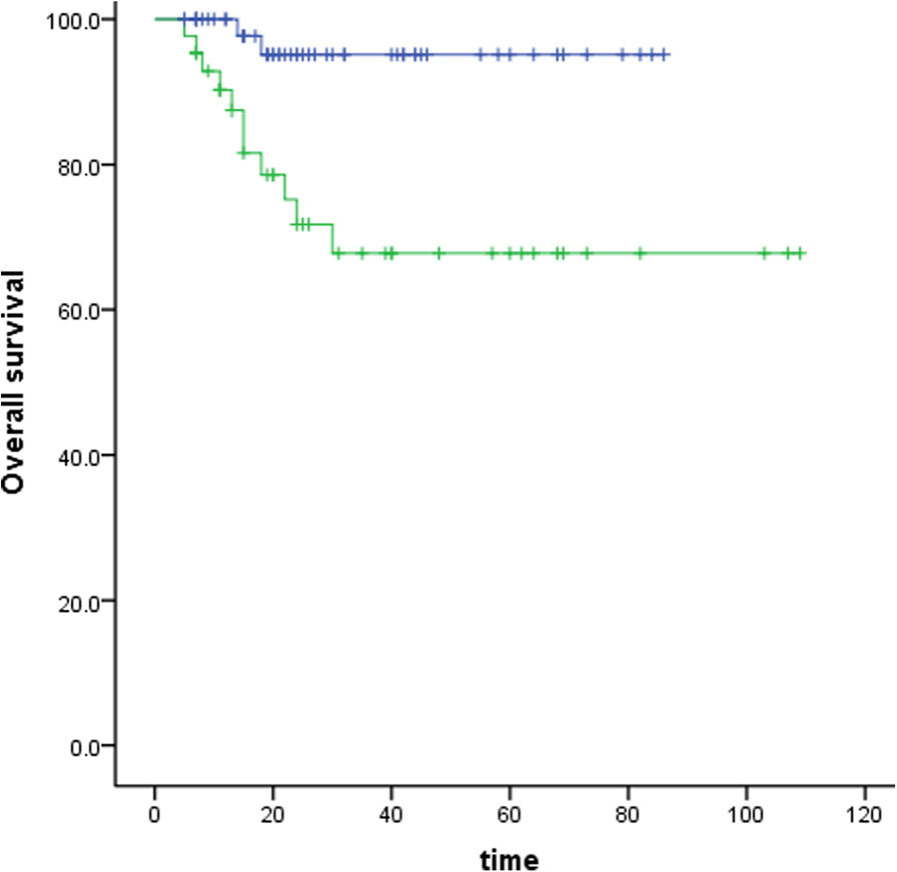
Effect of tumor size on overall survival (*P =* 0.00) of patients

No significant correlation with OS was identified for TNM stage, pathological classification, age, lymphovascular space invasion (LVSI), or deep stromal invasion. However, anemia was identified as a significant unfavorable OS factor (*P* = 0.00, Fig. 2), as well as lymph node (*P* = 0.04, Fig. 3) and tumor size (*P* = 0.00, Fig. 4). Similar correlation results were obtained for PFS (Table 2). We examined the aforementioned significant variables using multivariate analyses. Anemia and tumor size were also identified as significantly unfavorable factors for OS (*P* = 0.008, *P =* 0.045) (Table 3).

**Table 2:** Univariate OS and PFS analyses

Variables	Number	OS		PFS	
		*x* ^2^	*P* value	*x* ^2^	*P* value
Age					
≤ 40	8	1.04	0.307	2.12	0.15
> 40	90				
LVSI					
Yes	37	0.47	0.49	0.298	0.585
No	61				
Anemia before radiotherapy					
Yes	27	14.76	0.00*	7.17	0.01*
No	71				
Deep stromal invasion					
Yes	43	3.26	0.71	0.99	0.32
No	55				
PNI					
Yes	24	1.12	0.22	1.19	0.28
No	74				
LNM					
Positive	37	4.06	0.04*	5.41	0.02*
Negative	61				
Tumor size					
≥ 4 cm	43	9.22	0.00*	7.20	0.01*
< 4 cm	55				
Histology					
Squamous	91	0.00	0.96	0.52	0.47
Others	7				
TNM stage					
I–II	61	0.45	0.50	0.42	0.52
III	37				

*LNM* lymph-node metastasis, *PNI* perineural invasion, *LVSI* lymphovascular space invasion

*P < 0.05.

**Table 3:** Multivariate OS and PFS analyses

Variables	OS			PFS		
	*P* value	HR	95% CI	*P* value	HR	95% CI
Anemia before radiotherapy	0.008*	5.13	(1.54–17.04)	0.048*	2.59	1.01–6.65
Tumor size	0.045*	4.82	(0.67–6.48)	0.052	2.85	0.99–8.21
Lymph node positive	0.21	2.08	(1.03–22.49)	0.075	2.39	0.92–6.25

*HR* hazard ratio

*P < 0.05

### Acute toxicity

The detailed acute toxicities for the entire study population (*n* = 98) are listed in Table 4. The incidence of acute grade 3 rectal toxicity was of 1.02% (one patient). No grade 4 gastrointestinal toxicity occurred. The incidences of acute grade 3 and 4 hematologic toxicity were 17.35 and 1.02%, respectively. Additionally, one case of acute grade 3 bladder toxicity and two cases of acute grade 3 skin toxicity were identified.

**Table 4:** Acute toxicity for patients treated with RT

Grade	0	1	2	3	4
Category					
Rectum	59(60.20)	21(21.42)	17(17.34)	1(1.02)	–
Myelosuppression	22(22.40)	21(21.42)	37(37.76)	17(17.35)	1(1.02)
Upper gastrointestinal	43(43.88)	17(17.35)	38(38.78)	–	–
Skin	–	43(43.88)	53(54.08)	2(2.04)	–
Bladder	51(52.04)	37(37.76)	9(9.18)	1(1.02)	–

### Failure rate

At the time of analysis, treatment failure was observed in 18 patients (18.37%), including 10 patients 55.56(%) with pelvic recurrence, four patients (22.22%) with postoperative stump recurrence, two patients (11.11%) with lung metastasis, and one patient (5.56%) with brain metastasis while one patient (5.56%) died of adhesions. The time to recurrence ranged from 5.2 to 26.7 months (median of 11.3 months). Ten patients (10.20%) relapsed within 1 year of radiotherapy start, accounting for 55.56% of all treatment failures.

## Discussion

The poor prognosis factors for operable cervical cancer include positive margins, deep invasion, larger tumors, LVSI, and lymph node metastasis [7–12]. The results of this study showed that the 5-year OS and PFS were 82.0 and 76.0%, respectively. The frequency of grade 3 or more adverse reactions caused by the radiation was low. Furthermore, in patients for whom the overall therapeutic effect was satisfactory, there was no unacceptable side effect.

A lot of research on postoperative radiotherapy for cervical cancer worldwide has focused on the benefits of adjuvant radiotherapy, radiotherapy indications, and comprehensive radiotherapy and chemotherapy. Several of the early studies about adjuvant radiotherapy or chemoradiotherapy for cervical cancer revealed that postoperative radiotherapy or chemoradiotherapy could reduce the risk of tumor recurrence. In a phase III clinical study named GOG92, which enrolled 277 patients with multiple risk factors, 137 patients were given radiotherapy with 46 Gy (23 F) or 50.4 Gy (28 F), and the others were followed up regularly. The study found a significant reduction in relapse within the radiotherapy group (HR = 0.54, 90% CI 0.35–0.81, *p* = 0.007) and a decreased risk of death (HR = 0.58, 90% CI 0.40–0.85, *p* = 0.009). The GOG109 American study [4] enrolled 243 stage IA2, IB, and IIA cervical cancer patients who were randomly divided into two groups: 127 patients received chemoradiotherapy and 116 received radiotherapy alone. This study reported significant differences in the relapse and overall survival rates between the two groups (*P* = 0.003 and *P* = 0.007, respectively). Postoperative chemoradiotherapy reduced the 4-year PFS and OS by 17 and 10%, respectively, compared with the radiotherapy alone group. Song et al. [13] enrolled 110 cervical cancer patients who were divided into three groups: radiotherapy (1990–1999), radiotherapy (2000–2010), and chemoradiotherapy (2000–2010). They found that chemoradiotherapy reduced local recurrence (*P* = 0.012) and distant metastasis (*P* = 0.027).

Previous reviews have strongly suggested that concurrent chemotherapy and radiotherapy (CCRT) improves the OS and PFS rates in cervical cancer patients, regardless of whether platinum-based chemotherapeutic agents are used, with an absolute benefit of 10 and 13%, respectively [14, 15]. The National Comprehensive Cancer Network (NCCN) has established CCRT as the standard of treatment for cervical cancer in patients with high-risk factors. Based on previous studies [1, 3–6, 16], we used cisplatin alone in most of the patients enrolled in this study, totaling 71 cases (72.45%) with CCRT. However, the 5-year OS and PFS rates were lower than in previous studies. This difference may be attributable to the smaller sample size of our study, especially for the low number of patients followed beyond 5 years.

In this study, the age of the enrolled patients ranged from 29 to 76 years, with a median age of 52 years. It has been reported that patients younger than 35 years old were more susceptible to local recurrence or distant metastasis [17]. Kastritis [18] divided 218 patients into three groups according to age as follows: younger than 35 years old, between 35 and 70 years old, and older than 70 years old. They found that the median survival time in the younger group, median group, and older group was 9 months (95% CI 5.8–12), 14.5 months (95% CI 11–18), and 10 months (95% CI 6.9–13, *P* = 0.004), respectively. However, in our study, age was not identified as a prognostic factor for OS and PFS, possibly because of the small number of patients in the younger age group. Future studies in larger cohorts of patients will be necessary to validate this question.

Histology, FIGO stage, and deep stromal invasion were previously reported as prognostic factors for cervical cancer patients treated with RT [19–21]. In contrast, our study showed that these parameters did not affect the OS and PFS rates, which may be associated with the lower number of positive cases. Recently, perineural invasion (PNI) has attracted attention as a new prognostic factor in cancer patients. Therefore, we aimed to investigate the prognostic value of PNI in patients with cervical cancer. Cho et al. [22] showed that the significance of PNI as an independent predictor for prognosis was limited but was significantly associated with other prognostic factors. We did not reach the same conclusion with our patient cohort.

Lymph node status has been reported as a prognostic factor for cervical cancer [23]. Likewise, our study showed that lymph node status was a prognostic factor of OS (*P* = 0.04) and PFS (*P* = 0.02) by univariate analyses. However, multivariate analyses did not yield the same result. Regarding tumor size, a previous study [24] reported that tumor diameter was one of the main factors impacting local control by radiotherapy and prognosis. Likewise, we obtained a similar result of OS.

The lack of cellular oxygen is a known factor influencing the effects of radiation. Consequently, anemia may decrease tumor sensitivity to CCRT. Lee [25] found that anemia was an important indicator of poor prognosis or a sign of advanced disease, especially in pelvic tumor patients (*P* < 0.01). A retrospective analysis of 2454 cases of cervical cancer divided the patients into three groups according to their hemoglobin levels (9, 10, and 12 g/dl) based on the data obtained before and during the treatment. The researchers found that anemia was associated with disease recurrence, metastasis, and disease-specific survival rates (*P* < 0.001) [26]. Our study showed that anemic patients had significantly lower OS and PFS rates (*P* = 0.00 and *P* = 0.01, respectively). The same outcome was obtained using multivariate analyses (*P* = 0.008 and *P* = 0.048, respectively). Therefore, correcting an anemic condition before radiotherapy may improve OS and PFS.

The incidence of lung metastasis from cervical cancer is low, with an average incidence of 2.1–6.1% [27]. Ki [28] et al. retrospectively analyzed 56 cases of cervical cancer with lung metastases. They found that the median survival time was 12 months and that the average survival time was 40.7 months for patients with less than three metastases and 25 months for the others (*P* < 0.05). In our study, for patients who developed lung metastasis, the metastases appeared 5 months after the CCRT, followed by death 8 months later, which was significantly shorter than in the abovementioned report in terms of median survival time.

Blood metastases with cervical cancer are mainly found in the liver, lungs, and bones and rarely in the brain [29], which was reported to occur with an incidence of 0.4 to 1.2% [30]. Brain metastasis usually indicates a poor prognosis. Sato [31] reported the case of a cervical cancer patient who was diagnosed with brain metastases as the first symptom. Although the patient received CCRT, lung and liver metastasis rapidly appeared. The patient died 7 months after the brain metastases were identified. A retrospective analysis of 11 cases of cervical cancer patients with brain metastases showed that the small cell type was prone to metastasize to the lungs, which was a risk factor for cervical cancer brain metastases [32]. One patient developed brain metastases 11 months after the postoperative CCRT and died 2 months later.

Several studies have reported adverse reactions following postoperative radiotherapy. The frequency of these reactions is slowly declining, especially since the development of IMRT. Pu et al. [33] found a low incidence of approximately 5.6% of grade III and IV radiotherapy side effects in 285 cervical cancer patients who received CCRT. In our study, grade III and IV acute radiation enteritis was present in 1.02% of the patients, and only one patient died of adhesions. The incidence of grade I and II myelosuppression was 21.42 and 37.36%, respectively, and only one patient developed grade IV myelosuppression, which was found to be a symptom of the treatment. Similar to the above findings, we found 12 cases with more than three types of adverse reaction, accounting for 12.24% of the cases.

Some limitations of our study need to be addressed. First, the sample size was relatively small, and the follow-up time was slightly short (median of 37 months). In addition, because of its retrospective nature, potential important confounding biases might have been disregarded in the analysis, such as the selection bias introduced by the surgeons who determined which patients should be considered for CCRT. Moreover, the surgical and radiological techniques or histopathological diagnosis methods might have changed during the study period duration, which could have influenced our results. These factors can only be eliminated in a prospective randomized, controlled study.

## Conclusions

The clinical outcome from alternating chemoradiotherapy for high-risk cervical cancer patients, especially in those with highly advanced nodal disease, is promising in terms of both the sufficient efficacy level and the acceptably low toxicity level. However, perineural invasion was identified as another factor of poor prognosis, which should capture sufficient attention in the future.

## Abbreviations

CCRT: Concurrent chemotherapy and radiotherapy
LNM: Lymph node metastasis
PNI: Perineural invasion
